# Homogenisation of the Local Thermal Conductivity in Injection-Moulded Short Fibre Reinforced Composites

**DOI:** 10.3390/polym14163360

**Published:** 2022-08-17

**Authors:** Majid Mokarizadehhaghighishirazi, Bart Buffel, Stepan V. Lomov, Frederik Desplentere

**Affiliations:** 1 Research Group ProPoliS, Department of Materials Engineering, KU Leuven Campus Bruges, Spoorwegstraat 12, 8200 Bruges, Belgium; 2 SIM M3 Program, Technologiepark 48, 9052 Zwijnaarde, Belgium; 3 Department of Materials Engineering, KU Leuven, Kasteelpark Arenberg 44, Box 2450, 3001 Leuven, Belgium

**Keywords:** effective thermal conductivity, homogenisation, fibre orientation, micro-computed X-ray tomography, Autodesk Moldflow^®^ simulation

## Abstract

This paper deals with predicting the effective thermal conductivity (ETC) of injection-moulded short fibre reinforced polymers (SFRPs) using two different homogenisation schemes: a scheme based on the dielectric theory for pseudo-oriented inclusions and a two-step homogenisation model based on the mean-field homogenisation approach. In both cases, the fibre orientation tensor (FOT) obtained from Autodesk Moldflow^®^ simulation was used. The Moldflow FOT predictions were validated via structure tensor analysis of micro-computed X-ray tomography (micro-CT) scans of the part. In the dielectric-wise approach, the orientation of fibres was originally defined by a scalar parameter, which is related to the diagonal components of the FOT. In the two-step homogenisation approach, an interpolative model based on the Mori–Tanaka theory is used in the first step for calculating the ETC for the ideal case of unidirectional fibre alignment, followed by a second step in which orientation averaging based on the FOT inside each element is applied. The ETC was calculated using both schemes for the specific case of uniform fibre orientation distribution and at three different locations with non-identical FOTs of an injection-moulded SFRP part. The results are compared with each other and evaluated against the direct numerical simulation for the uniform fibre orientation and experimental measurements for the injection-moulded SFRP. This shows that while the two-step homogenisation can predict the ETC in the full range of orientations between the perfectly aligned and uniformly distributed fibres, the dielectric-wise approach is only capable of modelling the ETC when distributions are close to the two extreme ends of the orientation spectrum.

## 1. Introduction

Thanks to the large-scale manufacturing capacity of injection moulding, there has been dramatic growth in the production and applications of short fibre reinforced polymers (SFRPs) [1]. Within an injection-moulded SFRP part, however, the orientation of fibres may change considerably because of the non-uniform shear stress profile developed during injection [2]. This may result in anisotropic and heterogeneous mechanical, electric and thermal properties at different locations in injection-moulded SFRPs, which emphasises the importance and difficulties of developing efficient modelling approaches to predicting composite behaviour [3]. Thermal conductivity, which is an important parameter in the injection moulding simulations of SFRPs, has been regarded as a scalar constant in the existing software packages. Nevertheless, not only may it vary at different locations within injection-moulded SFRPs but it is a direction-dependent variable [4]. Therefore, thermal conductivity can be regarded as a second-order tensor that is influenced by the shape and orientation of fibres [5]. Since this can affect local cooling rates, part shrinkage and warpage, and hence the mechanical performance of the part, this study aimed to predict the effective thermal conductivity (ETC) by considering the local fibre orientation.

To obtain a homogenised property in heterogeneous media, one can choose either asymptotic or analytical-based homogenisation approaches [5]. If the complicated microstructure is provided in detail, asymptotic homogenisation using the finite element method (FEM) may predict the effective properties of composites rather precisely [6,7,8]. However, generating and meshing a representative volume element (RVE) that minutely describes the fibre’s placement and orientation in the injection-moulded SFRP is unfeasible. The computational process might be inefficient, time-consuming and costly [9]. Alternatively, analytical-based models can be used. For modelling the homogenised thermal conductivity of heterogeneous materials, two categories can be defined: Models based on the Eshelby theory of inclusions and methods based on Laplace’s heat transfer equation [10]. Regarding the latter, which is also called Maxwell’s homogenisation scheme [11], early works considered a dilute dispersion of ellipsoidal inclusions embedded in an isotropic matrix [12]. When the volume fraction of inclusions is high, differential methods based on the work of Bruggeman can be introduced to the workflow [13]. Using the Maxwell–Bruggeman solutions, Giordano [14] has proposed explicit formulae to predict the effective permittivity based on the dielectric theory of inclusions for unidirectional and uniform dispersions of ellipsoidal inclusions inside an isotropic matrix. Then, this model has been expanded to include all orientational distributions between the uniform and unidirectional ones by defining a scalar parameter that is dependent on the angular distribution of inclusions [15]. In this paper, the possibility of using Giordano´s model for predicting the ETC of injection-moulded SFRPs will be studied.

With regard to the models based on the Eshelby theory of inclusions, which refers to the mean-field homogenisation, a method that has become popular is two-step homogenisation [16]. The procedure for implementing this model starts with decomposing the RVE into sets of pseudo-grains in which the orientations of fibres are identical. Homogenisation takes place within each pseudo-grain in the first step using a mean-field model, followed by homogenisation amongst all pseudo-grains in the second step. The first step in the homogenisation is commonly carried out via Mori–Tanaka (MT) or Lielens double inclusion (DI) models, whereas Voigt or Reuss models are normally used in the second step [17]. For homogenising the thermal conductivity, it has been reported that the MT–Voigt combination will predict the ETC most accurately compared to other possibilities [18]. Although adopting this approach may result in accurate predictions of effective properties, there are two things to keep in mind. First, the RVE decomposition necessitates analysing the orientation distribution function (ODF). Extracting data from the ODF could be time-consuming and tedious. Alternatively, the fibre orientation tensor (FOT) is a more practical orientation descriptor, since it can be derived directly from orientation equations solved in simulation software packages [19]. Moreover, when an orientation tensor is calculated for an assembly with a certain ODF, and then the ODF is reconstructed using the orientation tensor, some information (higher harmonics) is lost and the ODF is changed [20]. The second consideration is the necessity of transforming the homogenised tensorial property from the local coordinate system defined in each pseudo-grain to the global coordinate of the RVE [21].

It is possible to develop a more straightforward two-step homogenisation model which could be equivalent to what has been explained above, yet with fewer complexities. In the first step, a unit cell with a simple arrangement of unidirectional fibres with the same fibre volume fraction as in the original RVE is assumed. The unit-cell can be homogenised via different analytical models or the FEM. In the second step, an orientation averaging rule is used to take the fibre misalignment into account [22]. To the best of our knowledge, the efficiency of this method has already been demonstrated in the homogenisation of elastic properties [23], but not for the ETC prediction. Since the Voigt assumption is preferred in the second step of the ETC homogenisation [18], the very early orientation averaging model proposed by Advani and Tucker [24], which provides orientation averaged properties using the FOT and associated unidirectional properties by assuming the Voigt interaction is used, in this study. The ETC of the composite can thus be predicted without having to worry about the ODF and tensor transformation analyses.

**Figure 1:**
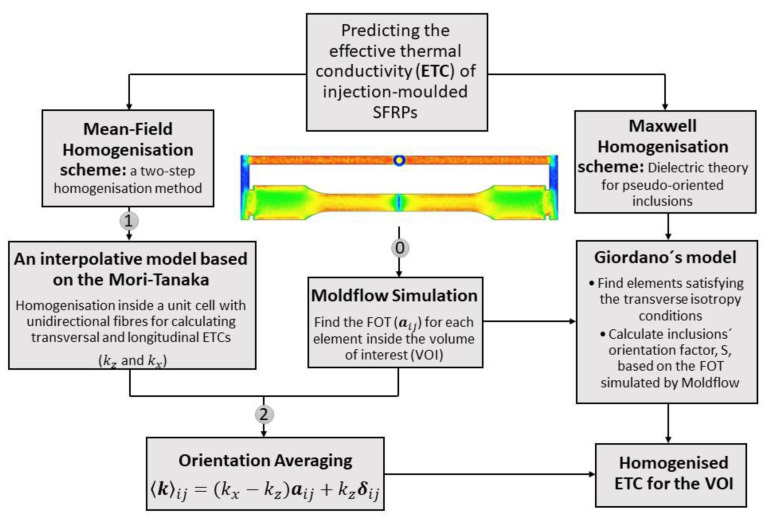
The schematic representation of the developed simulation-based homogenisation approaches for predicting the ETC of injection-moulded SFRPs.

Although various models have been developed to predict the ETC of multi-oriented inclusion-reinforced composites [5], developing an efficient method for predicting the ETC of injection-moulded SFRPs has been less investigated. This is explicitly addressed in this paper by comparing the developed two-step homogenisation scheme to the reformulated Giordano´s model [15], as illustrated in Figure 1. Autodesk Moldflow^®^ is used to find the FOT which is required in both approaches. It has been shown that injection moulding software packages such as Autodesk Moldflow^®^ and Moldex3D^®^ can accurately predict the actual fibre orientation distribution in injection-moulded parts [25,26]. However, in this study the FOT obtained from the Moldflow simulations is also evaluated against experimental measurements of the injection-moulded part, performed via micro-computed X-ray tomography (micro-CT) scans. As mentioned previously, fibre orientation in Giordano´s model is described by a scalar parameter whose relationship with the components of the FOT has not been stated explicitly. This will be clarified in this research by three basic equations. Furthermore, a two-step homogenisation method using orientation averaging is developed to find the local ETC of injection-moulded SFRPs. In the first step, an interpolative model based on the MT model is used for calculating the ETC in the hypothetical case of perfectly aligned fibres. The results are compared to those obtained from Mori–Tanaka, simplified Giordano, and FEM analyses, the latter which is supposedly the exact solution. In the second step, the actual misaligned fibre orientation inside each element (FOT on elements) is taken into account using an orientation averaging approach. For validation, the results are evaluated against the experimental measurements and FEM homogenisation for two different SFRP systems.

## 2. Methodologies

### 2.1. Homogenisation Models

#### 2.1.1. Dielectric Theory for Pseudo-Oriented Inclusions: Giordano’s Model

Within the model proposed by Giordano [15], which was originally developed for predicting effective electric permittivity, Maxwell´s solution is expanded based on Bruggeman’s differential approach to consider the orientational order/disorder of ellipsoidal particles in a composite by introducing a scalar parameter (S). The main results which have been reformulated to calculate the longitudinal ($k_{xx}$) and transversal ($k_{zz}$) ETC of SFRPs are

(1) $$1-\nu_{f}=\frac{k_{f}-k_{zz}\;}{k_{f}-k_{m}}{\left(\frac{k_{m}}{k_{zz}}\right)}^{\frac{3L\left(1-2L\right)}{2-3L+S-3SL}}{\left[\frac{\left(1+3L-S+3SL\right)k_{m}+\left(2-3L+S-3SL\right)k_{f}}{\left(1+3L-S+3SL\right)k_{zz}+\left(2-3L+S-3SL\right)k_{f}}\right]}^{\frac{{\left(1-3L\right)}^{2}\left(2+S\right)\left(1-S\right)}{\left(2-3L+S-3SL\right)\left(1+3L-S+3SL\right)}},\;$$

(2) $$1-\nu_{f}=\frac{k_{f}-k_{xx}\;}{k_{f}-k_{m}}{\left(\frac{k_{m}}{k_{xx}}\right)}^{\frac{3L\left(1-2L\right)}{2-3L-2S+6SL}}{\left[\frac{\left(1+3L+2S-6SL\right)k_{m}+\left(2-3L-2S+6SL\right)k_{f}}{\left(1+3L+2S-6SL\right)k_{xx}+\left(2-3L-2S+6SL\right)k_{f}}\right]}^{\frac{2{\left(1-3L\right)}^{2}\left(2S+1\right)\left(1-S\right)}{\left(2-3L-2S+6SL\right)\left(1+3L+2S-6SL\right)}}.\;$$

In Equations (1) and (2), $\nu_{f}$, $k_{m}$ and $k_{f}$ are the volume fraction of fibres, the thermal conductivity of the matrix and thermal conductivity of fibres, respectively. L is called the depolarisation factor, which is dependent on the aspect ratio of fibres (fibre length divided by its diameter, e=l/d), which can be computed from

(3) $$L=\frac{e}{4{\left(\sqrt{e^{2}-1}\right)}^{3}}\left[2e\sqrt{e^{2}-1}+ln\frac{e-\sqrt{e^{2}-1}}{e+\sqrt{e^{2}-1}}\right],\;for\;e>1,$$

The depolarisation factor is calculated for an ellipsoidal inclusion with $a_{x}>a_{y}=a_{z}$, where $a_{x}$, $a_{y}$ and $a_{z}$ are semi-axes of the ellipsoid. Another assumption in developing the S parameter in this model is that the thermal conductivity of the composite is assumed to be transversely isotropic and can be represented by a tensor k:

(4) $$\left[k\right]=\left[\begin{array}{ccc} k_{xx} & 0 & 0\\ 0 & k_{zz} & 0\\ 0 & 0 & k_{zz} \end{array}\right].$$

Concerning the orientational factor S, it can be shown that it is related to the diagonal components of the FOT ($a_{ij}$), as in

(5) $$S=(3a_{xx}-1)/2=1-3a_{yy}=1-3a_{zz}$$

When S=1, the state of complete order is considered; i.e., all fibres are assumed to be aligned with x-direction ($a_{xx}=1$). In this case, Equations (1) and (2) will be simplified to calculate the ETC in the case of complete order (unidirectional fibre alignment):

(6) $$1-v_{f}=\frac{k_{f}-k_{z}}{k_{f}-k_{m}}{\left(\frac{k_{m}}{k_{z}}\right)}^{L},\;$$

(7) $$1-v_{f}=\frac{k_{f}-k_{x}}{k_{f}-k_{m}}{\left(\frac{k_{m}}{k_{x}}\right)}^{1-2L}.$$

The case S=0 refers to a random orientation in which fibres have no preferential direction ($a_{xx}=a_{yy}=a_{zz}=1/3)$. The deviation from the complete ordered condition increases as S takes values from 1 to 0.

It should be noted that assuming the transverse isotropic behaviour requires two conditions to be satisfied in the FOT; $a_{yy}=a_{zz}$ and $a_{ij}=0\;when\;i\neq j$; that is, the off-diagonal components of the orientation tensor are zero. This leads to the major limitation of Giordano´s model: Some particular distributions of fibres were considered, which are not exhaustive of all possible existing statistical distributions. In other words, there can be some distributions of inclusions, or equivalently FOT on some elements that cannot be represented by the parameter S. In this paper, we will investigate whether this limitation can cause major restrictions in predicting the local ETC of injection-moulded SFRPs.

#### 2.1.2. The Mori–Tanaka (MT) Model and Lielens Interpolation

A family of models for predicting the effective properties of composite materials with the no-dilute concentration of inclusions has been developed from a proposal originally made by Mori and Tanaka [27], which is here reformulated for predicting the ETC of SFRPs.

The ETC of the composite can be expressed as a second-order tensor, k, which can be calculated from

(8) $$k=k_{m}+v_{f}\left(k_{f}-k_{m}\right)A,$$

where A is the intensity-concentration tensor (similar to the strain-concentration tensor in elasticity), which relates the average temperature gradient (or strain) of fibres to that of the composite. An alternative concentration tensor, $\hat{A}$, is defined so as to link the average temperature gradient (or strain) of fibres to that of the matrix. The relationship between A and $\hat{A}$ is

(9) $$A=\hat{A}{\left[\left(1-v_{f}\right)I+v_{f}\hat{A}\right]}^{-1},$$

where I is the second-order identity tensor. Different models in the mean-field homogenisation scheme have suggested various formulae for obtaining the concentration tensors. Based on the MT, which is equivalent to the lower bound solution, we have:

(10) $${\hat{A}}^{MT}={\hat{A}}^{lower}={\left[I+Sk_{m}^{-1}\left(k_{f}-k_{m}\right)\right]}^{-1}.$$

S is called the interior-point Eshelby thermal conductance tensor, which is a diagonal tensor depending only on the shape of inclusions. However, for the case of an ovary or elongated ellipsoidal inclusion with semi-axes $a_{x}>a_{y}=a_{z}$, the diagonal components of S can be computed from [28]:

(11) $$S_{xx}=1-2S_{yy},$$

(12) $$S_{yy}=S_{zz}=\frac{a_{z}^{2}a_{x}}{2{\left(a_{x}^{2}-a_{z}^{2}\right)}^{3/2}}\left\{\frac{a_{x}}{a_{z}}{\left(\frac{a_{x}^{2}}{a_{z}^{2}}-1\right)}^{1/2}-\cosh^{-1}\left(\frac{a_{x}}{a_{z}}\right)\right\}.$$

The inverse Mori–Tanaka (IMT) formulation, which is identical to the upper bound solution, can be derived by assuming that elliptical inclusions of the polymer matrix are embedded in a continuous medium of the fibre material, resulting in a concentration tensor:

(13) $${\hat{A}}^{IMT}={\hat{A}}^{upper}=\left[I+Sk_{f}^{-1}\left(k_{m}-k_{f}\right)\right].$$

As the MT prediction of the effective properties (the lower bound) may deviate from true values at high fibre volume fractions, Lielens et al. [29] proposed a formula to interpolate between the upper and lower bounds:

(14) $${\hat{A}}^{Lielens}={\left[\left(1-f\right){\left({\hat{A}}^{lower}\right)}^{-1}+f{\left({\hat{A}}^{upper}\right)}^{-1}\right]}^{-1}.$$

f is the interpolating factor which can be obtained from the fibre volume fraction, as shown in Equation (15). As a result, the prediction of the effective properties is improved at higher fibre contents.

(15) $$f=\left(v_{f}+v_{f}^{2}\right)/2.$$

#### 2.1.3. FEM Analysis: Unidirectional Fibre Alignment

**Figure 2:**
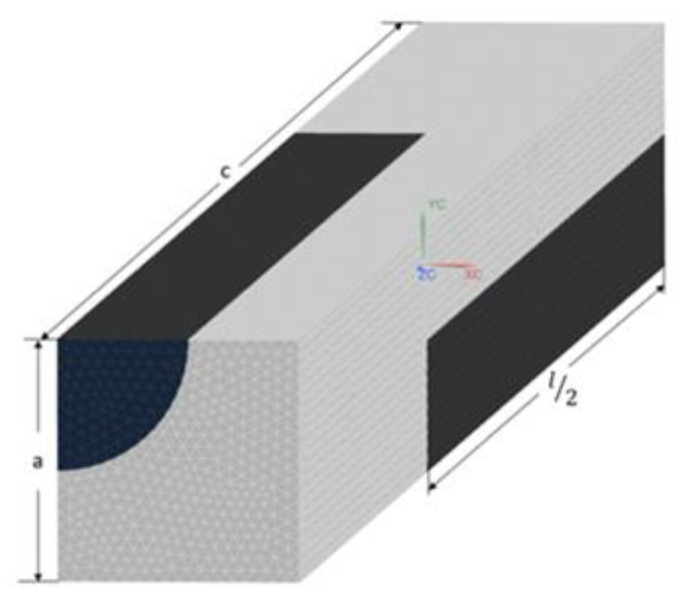
Representation of the RVE used for FEM modelling of the ETC in the case of unidirectional fibre alignment.

Experimental validation of the predicted ETC in the case of complete order is problematic because fabricating samples with perfectly aligned short fibres is unfeasible. Instead, the FEM can be used for calculating the supposedly exact solution for this case. Based on [27], staggered arrays with square packing were chosen as the repetitive volume element, which is illustrated in Figure 2. Dimensions of the unit cell (a and c) are calculated using the volume fraction of the inclusions ($v_{f}$) and the fibre´s length (l) and diameter (d). It should be noted that as long as we are dealing with unidirectional short fibres, increasing the size of the RVE to contain more fibres is useless, as it has been shown that increasing the size of the RVE, in this case, has almost no effect on the convergence of the values associated with the studied homogenised property [23].

For the ETC calculation, the steady-state heat transfer equation is considered. Boundary conditions are imposed on the RVE as follows: For obtaining the ETC along each direction (conduction axes), a fixed temperature constraint is imposed on the faces of the RVE, whose normal vector is parallel to that direction. The other four faces of the unit cell are insulated; i.e., zero heat flux condition is assumed on the faces orthogonal to the conduction axis. The FEM solution provides the average heat flux along the conduction axis ($Q_{i}$). Knowing the imposed temperature gradient ($\nabla T_{i}$) and on the basis of Fourier´s constitutive equation, the ETC along each axis can be computed using $k_{i}=\left|Q_{i}/\nabla T_{i}\right|$.

#### 2.1.4. Orientation Averaging

In order to incorporate the effect of fibre orientation on the ETC, consider k(p) as a tensorial property of the composite associated with a unidirectional microstructure aligned in the direction of p, which is a unit vector along each fibre´s axis. The orientation average of k is denoted by k and is defined by

(16) k=∮k(p)ψ(p)dp,

where ψ(p) is the ODF.

k must be a transversely-isotropic tensor with p as its axis of symmetry. Assume k(p) to be a second-order tensor, so that it can be expressed as:

(17) $$k\left(p\right)=\left[\begin{array}{ccc} k_{x} & 0 & 0\\ 0 & k_{z} & 0\\ 0 & 0 & k_{z} \end{array}\right].$$

On the other hand, this tensor must also have the form:

(18) $$k_{ij}\left(p\right)=cp_{i}p_{j}+d\delta_{ij},$$

where c and d are two scalar constants, and $\delta_{ij}$ is the Kronecker delta. If we take the orientation average of the property, ${\langle k\rangle }_{ij}$, we get:

(19) $${\langle k\rangle }_{ij}=c\langle p_{i}p_{j}\rangle +d\langle \delta_{ij}\rangle =ca_{ij}+d\delta_{ij.}$$

In other words, the orientation average of a second-order tensor is completely determined by the second-order orientation tensor, and by the underlying unidirectional property tensor [24]. Now we aim to find the relationships between the constants c and d, and unidirectional properties ($k_{x}$ and $k_{z}$). For this purpose, the first and second tensor invariants ($I_{1}$ and $I_{2}$) of two expressions of k based on Equations (17) and (18) are used to form a system of equations:

(20) $$I_{1}=trace\left(k\right)=k_{x}+2k_{z}=c+3d,$$

(21) $$I_{2}=1/2\left[{\left(trace\left(k\right)\right)}^{2}-trace\left((k^{2}\right))\right]=2k_{x}k_{z}+k_{z}^{2}=2cd+3d^{2}.$$

Solving the system of equations results in:

(22) $$d=k_{z}\;and\;c=k_{x}-k_{z}.$$

Substituting Equation (22) in Equation (19) results in Equation (23), in which the orientation average of the second-order tensorial property of SFRPs (for example, the ETC) can be calculated using the FOT and properties associated with unidirectional fibre alignment:

(23) $${\langle k\rangle }_{ij}=\left(k_{x}-k_{z}\right)a_{ij}+k_{z}\delta_{ij}.$$

### 2.2. Autodesk Moldflow Simulation

The injection moulding process was simulated via Autodesk Moldflow^®^ 2019 using 3D meshing. Fibre orientation tensors on elements were exported from the software to be used in homogenisation models. The FOT on elements was calculated using the Moldflow rotational diffusion model with auto-calculated fibre interaction coefficient and coefficients of asymmetry. It has been reported that the simulated FOT is dependent on the mesh size, and by mesh refinement, the simulated FOT may converge to the experimentally measured FOT [30]. In our simulations, the mesh size gradually decreased, until the mesh sensitivity became negligible.

### 2.3. Cases to Evaluate the Models

#### 2.3.1. Case study 1: Uniform Distribution of Fibres

The FEM-homogenised ETC of a composite reinforced with 10 volume percent randomly distributed cylindrical fibres with an aspect ratio of 10 (l=50 μm and d=5 μm) was taken from [18]. The matrix and fibres were assumed to be isotropic with thermal conductivities of 72.0 and 32.0 ${\mathrm{Wm}}^{-1}K^{-1}$, respectively. In order to predict the ETC of the composite with a uniform distribution of fibres via FEM homogenisation, the authors of [18] generated an RVE with the size of 100 μm^3^ using the random sequential absorption (RSA) algorithm. After imposing periodic boundary conditions and solving the Fourier´s constitutive equation numerically, they reported the ETC of 67.4 ${\mathrm{Wm}}^{-1}K^{-1}$ when the perfect interface was assumed between fibres and the matrix. This case was modelled using our two-step homogenisation mode and Giordano’s approach, and the results are compared to those of FEM homogenisation.

#### 2.3.2. Case Study 2: Fibre Orientation of Injection-Moulded SFRPs

DOMAMID 6LVG50H2BK, a commercial grade polyamide 6 (PA6) reinforced with 50 weight-percent short glass fibre (which is equivalent to around 30.8 volume percent), was injection-moulded to produce dog-bone shaped samples originally intended for tensile testing based on the ISO 527 1A standard. The part geometry and the injection moulding process parameters are represented in the Supplementary Material (Figure S1 and Table S1).

**Figure 3:**
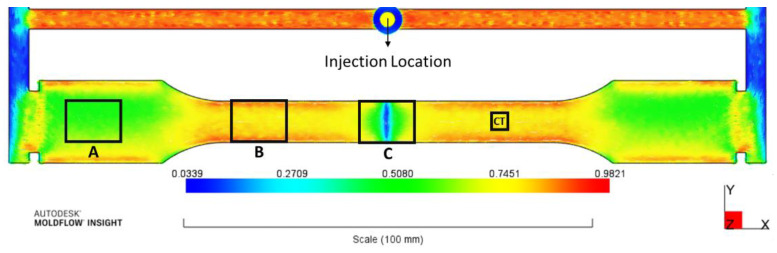
Variation of the first component of the FOT ($a_{xx}$) throughout the injection-moulded part based on the Autodesk Moldflow^®^ simulation. Three regions, A, B and C, were selected for evaluating the models against experimental measurements of the ETC. A was cut from the gauge length for the micro-CT analysis.

Thermal conductivity of unfilled PA6 was measured at 0.26 ${\mathrm{Wm}}^{-1}K^{-1}$, and the conductivity of glass fibres was assumed to be 1.04 ${\mathrm{Wm}}^{-1}K^{-1}$ [31]. The average length and diameter of glass fibres after injection moulding were found to be 260.8 and 11.4 μm, respectively, via micro-CT scans; Therefore, an average aspect ratio of around 23 can be considered for the glass fibres [32]. The 4 mm-thick tensile sample was injection-moulded via two gates located at two ends of the dog-bone sample in order to produce a weld-line at the middle of the part. This was done to make three zones in the injection-moulded sample in which the simulated FOTs would differ noticeably from each other, as illustrated in Figure 3. These regions are listed as A, B and C in Figure 3 corresponding to the wider section, gauge length and weld-line regions of the specimen, respectively. The detailed FOT analysis at these positions is discussed in Section 3.4.1. The flow direction was along the x-axis, and the thickness of the part was aligned with the z-axis. The local ETC was calculated using the two homogenisation approaches at three regions of the injection-moulded part and was evaluated against experimental measurements for assessment of the models.

### 2.4. Micro-Computed X-ray Tomography

Micro-CT scanning acquisition was performed using a GE Phoenix Nanotom system which was equipped with a 180 kV/15 W nanofocus X-ray tube and a 2300 × 2300 pixel detector on a 12-bit Hamamatsu flat panel. The X-ray tube’s accelerating voltage and the beam current were 100 kV and 135 μA, respectively. The installed target material consisted of tungsten on CVD synthetic diamond. There were no additional filter materials applied. The detector integration time was set to 0.5 s, and the number of projections was 3500. The specimen for micro-CT imaging was cut from the gauge length of the injection-moulded tensile bar, as illustrated in Figure 3, with dimensions of 4 × 4 × 4 mm^3^. The scans have a resolution of 2.5 μm per pixel, and the average glass fibre diameter was 11.4 μm. Reconstruction of scans was performed in Datos|x software, and micro-CT images were imported into the VoxTex to extract the fibre orientation tensor in the scanned region. The structural tensor analysis was then used to assign local fibre direction data to the components of the voxel model [33]. The process for creating voxel models from the X-ray computed tomography data was thoroughly explained in [34]. Further details for the fibre orientation analysis in VoxTex are presented in Section 3.4.1.

### 2.5. Experimental Measurement of the ETC

A contact-based transient method using the C-therm Trident instrument was selected to measure the thermal conductivity of injection-moulded samples along different directions at room temperature. The Modified Transient Plane Source (MTPS) sensor was used to carry out the measurements according to ASTM D7984. The single-sided MTPS sensor was equipped with a guard ring to facilitate one-dimensional heat transfer inside the sample, which is required for the thermal conductivity measurement of anisotropic materials. When the density (ρ) and specific heat ($C_{p}$) of the test sample are known, the thermal conductivity can be calculated via Equation (24) using thermal effusivity (ε), which is directly measured by the MTPS sensor:

(24) $$k=\varepsilon^{2}/(\rho C_{p}).$$

The specific heat of the material was determined at 1140 ${\mathrm{Jkg}}^{-1}K^{-1}$ at room temperature using a Q200 TA instrument differential scanning calorimeter (DSC), and the density was taken from the material supplier at 1560 ${\mathrm{kgm}}^{-3}$.

**Figure 4:**
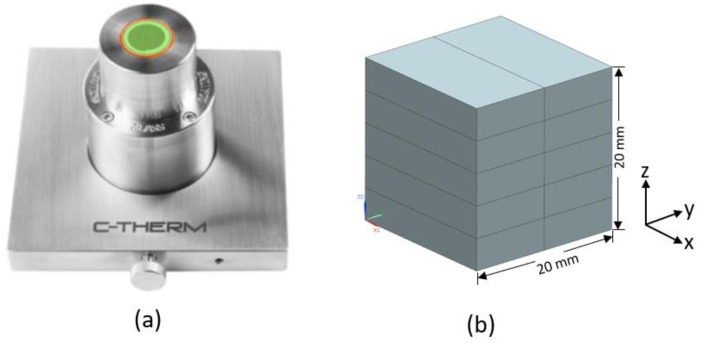
(**a**) The MTPS sensor, and (**b**) sample preparation for the ETC measurement.

As the MTPS sensor requires specimens with a minimum diameter of 18 mm to cover the sensor’s surface, samples with dimensions of 18 × 10 × 4 mm^3^ were milled at three different locations of the injection-moulded sample shown in Figure 3. For each region, 10 samples were polished and then glued to each other using a thin layer of epoxy paste in order to form a block of 18 × 20 × 20 mm^3^, as illustrated in Figure 4. Two blocks were made for each region. This allowed us to measure thermal conductivities along each axis by rotating this block on the sensor. For instance, if the x–y face of the block was in contact with the sensor, thermal conductivity along the z-axis (thickness) was measured. Three measurements were performed at room temperature for each face of the block, and the average value is reported as the ETC along each direction. Glycol was used as the contact agent between the sample and the sensor.

## 3. Results and Discussions

### 3.1. Calculation of the Depolarisation Factor and Eshelby Tensor for the Cases Studies

Looking at Equations (3) and (12) shows that the depolarisation factor is identical to the second (or third) diagonal component of the Eshelby tensor. This can be proven by assuming $e=a_{x}/a_{z}$ and some other mathematical simplifications. Therefore:

(25) $$L=S_{yy}=S_{zz}=\frac{e^{2}}{2\left(e^{2}-1\right)}-\frac{eln\left(e+\sqrt{e^{2}-1}\right)}{2{\left(e^{2}-1\right)}^{\frac{3}{2}}}.$$

Originally, the depolarisation factor L used in Equations (1) and (2) is a simplified notation of three depolarisation factors, $L_{x}$, $L_{y}$ and $L_{z}$, defined along each semi-axis of the ellipsoidal inclusion. Derivation of these depolarisation factors is explicitly expressed in terms of elliptic integrals in the reference [14], where it has been shown that $L_{x}+L_{y}+L_{z}=1$. However, for an ellipsoid of rotation in which $a_{y}=a_{z}$, we have $L_{y}=L_{z}=L$ and $L_{x}=1-2L$. This is exactly analogous to the concept of the Eshelby tensor, in which for the same inclusion we have$\;S_{yy}=S_{zz}$ and $S_{xx}=1-2S_{yy}$. This means that the depolarisation vector corresponds to the diagonal Eshelby tensor, both having two independent variables. Therefore, the construction of the Eshelby tensor for the electric/thermal problem is completely equivalent to the formulation based on the depolarisation factors, which was developed quite earlier in the literature [35]. The formalism based on the Eshelby tensor is simply more modern and useful for some generalisations, for instance, when the matrix properties are anisotropic. It is also interesting to notice that in Equation (25) $\underset{e\to \infty }{\lim}L=\underset{e\to \infty }{\lim}S_{22}=1/2$; that is, for fibres with high aspect ratios, we have:

(26) $$L=0.5,\;S=\left[\begin{array}{ccc} 0 & 0 & 0\\ 0 & 0.5 & 0\\ 0 & 0 & 0.5 \end{array}\right].$$

**Figure 5:**
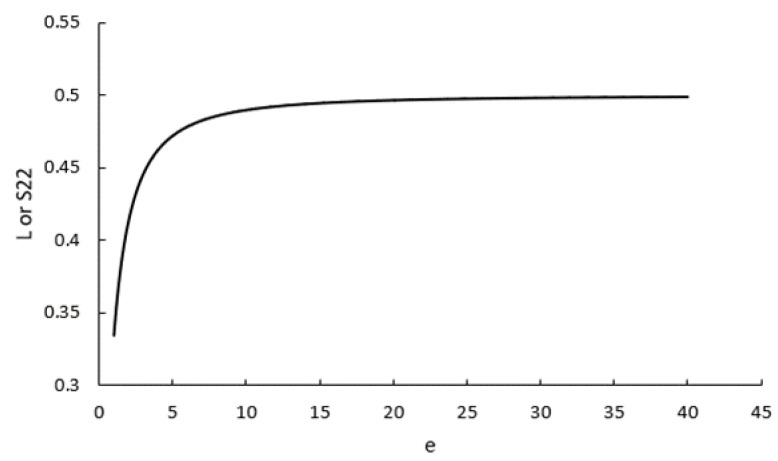
The variation of the depolarisation factor, which is equivalent to the second (third) diagonal component of the Eshelby tensor, versus the aspect ratio of fibres.

To understand what a “high aspect ratio” implies, Figure 5 shows how L or $S_{yy}$ changes with different values of aspect ratio (e). It can be seen that the curve reaches its limit at 0.5 rather quickly. For example, for the composites of cases 1 and 2 with aspect ratios of 10 and 23, $L=S_{yy}=0.490$ and 0.497, respectively. As a result, in both cases, Equation (26) was a reasonable approximation for further calculations.

### 3.2. ETC Prediction for Unidirectional Fibre Alignment

In the first phase of the two-step homogenisation approach, the ETC of the aforementioned case studies for unidirectional fibre alignment was obtained using Giordano’s model (Equations (6) and (7)), the Mori–Tanaka model, and the Lielens interpolation, as described in Section 2.1.2. Unidirectional fibre reinforced composites are transversely isotropic; so the ETC is defined by longitudinal and transversal components, denoted by $k_{x}$ and $k_{z}$ respectively. In Table 1, the results are compared with each other and evaluated against the FEM homogenisation. Table 1 indicates that there is no difference in predicted $k_{x}$ based on three models, which is also consistent with the FEM results. On the other hand, the ETCs perpendicular to the fibre´s direction are not identical.

**Figure 6:**
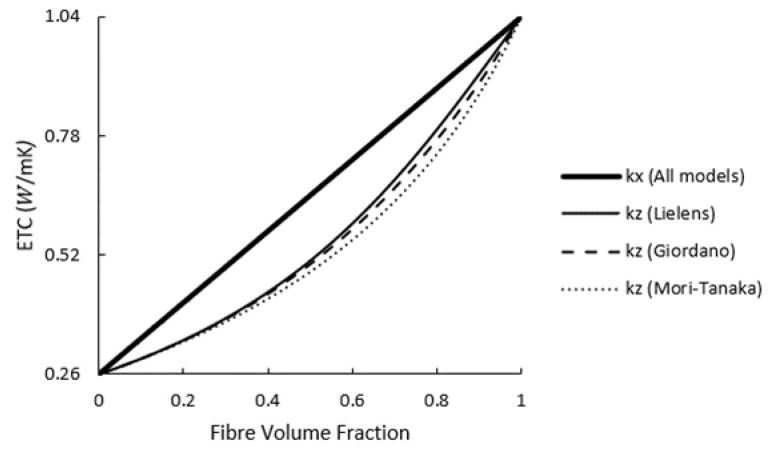
The variations of longitudinal and transversal thermal conductivities versus the fibre volume fraction for unidirectional short glass fibre reinforced PA6 obtained from Giordano, Mori–Tanaka and Lielens interpolation models.

In case study 2, where the composite contained a relatively high fraction of fibres, Giordano and Lielens predictions turned out to be the most compatible with the FEM homogenisation, whereas all models produced similar results in case study 1 with lower fibre content. $k_{x}$ and $k_{z}$ predicted by three models are plotted versus the fibre volume fraction for PA6 composites reinforced with unidirectional short glass fibres (aspect ratio of around 23) in Figure 6. As can be seen, while all models predict the linear fluctuations of $k_{x}$ in accordance with the Voigt upper bound approach, this is not the case for $k_{z}$ over the whole range of $v_{f}$. For low concentrations of fibres, all models provide the same predictions; but by increasing $v_{f}$, they start to deviate from each other. Nevertheless, good agreement is shown in transversal conductivities obtained from the Giordano and Lielens models up to $v_{f}=0.5$. For consistency, the data from Lielens interpolative model were hence taken to be used in the second step of the two-step homogenisation method.

### 3.3. ETC Prediction for the Uniform Distribution of Fibres: Case Study 1

In this section, the FEM homogenisation result of case study 1 taken from [18] is compared to that obtained from the two-step homogenisation and Giordano´s model. For uniform distribution of fibres, the orientation tensor $a_{ij}=\left[\begin{array}{ccc} 1/3 & 0 & 0\\ 0 & 1/3 & 0\\ 0 & 0 & 1/3 \end{array}\right]$ is considered, which is equivalent to S=0 in Giordano´s approach. Substituting S=0 into Equations (1) and (2) results in a simplified Giordano´s formulation where $k_{xx}=k_{zz}=k$:

(27) $$\left(1-v_{f}\right)\left(k_{f}-k_{m}\right)=\left(k_{f}-k\right){\left(\frac{k_{m}}{k}\right)}^{\frac{3L\left(1-2L\right)}{\left(2-3L\right)}}{\left[\frac{\left(1+3L\right)k_{m}+\left(2-3L\right)k_{f}}{\left(1+3L\right)k+\left(2-3L\right)k_{f}}\right]}^{2{\left(3L-1\right)}^{2}/\left(2-3L\right)\left(1+3L\right)}.$$

Solving Equation (27) by substituting the composite properties of case study 1 and L=0.49, calculated in Section 3.1, results in $k=67.09\;{\mathrm{Wm}}^{-1}K^{-1}$. Meanwhile, following the two-step homogenisation approach for this case, based on Equation (23) and Table 1, leads to an identical outcome of $k=67.10\;{\mathrm{Wm}}^{-1}K^{-1}$. Additionally, there is good agreement between these results and those of FEM-homogenised ET reported in [18]: $k=67.40\;{\mathrm{Wm}}^{-1}K^{-1}$. Therefore, it can be concluded that the two-step homogenisation method and the reformulated Giordano model could accurately predict the ETCs of composites in cases of unidirectional and uniformly distributed fibres.

### 3.4. ETC Prediction for Misaligned Fibres in Injection-Moulded SFRPs: Case Study 2

#### 3.4.1. Simulated FOT Predictions and Validation

In order to predict the ETCs of injection-moulded SFRPs, the misaligned fibre orientations were scrutinised at three zones of an injection-moulded tensile bar via Autodesk Moldflow^®^ simulations, as illustrated in Figure 3. First, the FOTs on elements associated with each region were extracted and analysed to explain the differences in fibre orientation at these positions. Region A, which was located after the gate in the wider section of the tensile bar, showed a fairly misaligned in-plane orientation, a typical fibre orientation distribution in injection-moulded parts. While the average $a_{xx}$ in this region was around 0.63, a significant proportion of fibres were still aligned with the y-axis, the average of $a_{yy}$ being 0.35. As expected, a very small number of fibres tended to be out of plane, along z-direction in this region.

**Figure 7:**
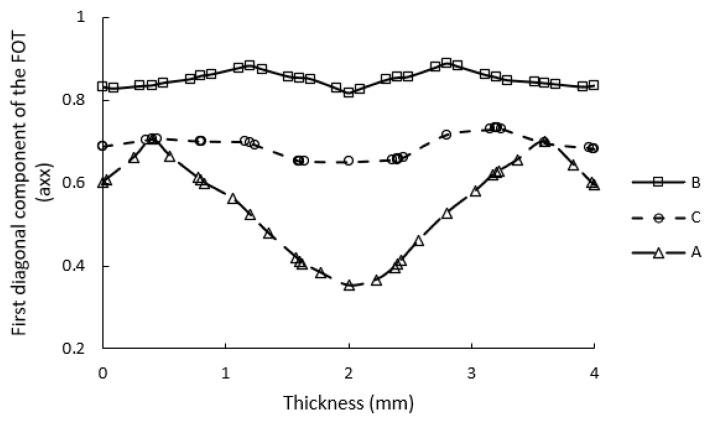
Variation in $a_{xx}$ throughout the thickness at various locations in the injection-moulded part. While the skin–shell–core structure is seen outside of the gauge length (region A), the fibre orientation throughout the thickness inside the gauge length is almost constant (regions B and C).

In contrast, due to converging flows in the gauge length of the sample, strong fibre alignment along the flow direction was seen in region B (average$\;a_{xx}=0.84$). Moreover, a small number of fibres were aligned along other two axes. Unlike these two samples, a considerable number of fibres were oriented out of the flow plane in region C because of the presence of a weld-line: on average,$\;a_{zz}=0.20$. The variation in $a_{xx}$ throughout the thickness of the injection-moulded part is shown in Figure 7. Despite the fluctuations in $a_{xx}$ throughout the thickness in region A, which are consistent with the skin–shell–core structure induced by the fountain flow in injection moulding [2], $a_{xx}$ in the gauge length of the samples remained almost unchanged due to the pronounced extensional flow.

For validating the simulated FOT results generated by Autodesk Moldflow^®^, experimentally measured FOT was obtained in the gauge length of the sample (outside of the weld-line region) through processing the micro-CT scans in the VoxTex software [34]. The micro-CT scans were imported to the software in the form of an image stack to set up a 3D model, the so-called voxel model. This is a 3D mesh of volume elements with the orientation vector and additional variables attached to each element. The next step was pre-processing the scans, which was performed by expanding the dynamic grey-scale range of the images and increasing the contrast by selecting the main part of the histogram. Then, a region of interest (ROI) with dimensions of 3125 × 3125 × 3125 μm^3^ was cut from the original micro-CT scans in the VoxTex.

For generating the voxel model, two important parameters should be defined. The first one is the mesh density, which determines the size of the voxel model. Our voxel model was produced by setting the mesh density to 20 pixels, resulting in 226,981 voxels in the ROI. The second parameter is the window radius, which determines the computation accuracy of the voxel model. The general rule is that the larger the diameter of the fibre in the image (in pixels), the larger the window size should be set. For our case, the window radius was set to 8.

Each voxel in the model has data about the average grey scale (denotes material density), degree of anisotropy and its principal direction. When performing fibre orientation analysis in VoxTex, voxels containing exclusively the matrix material must be excluded. For this purpose, an anisotropy histogram of the voxel model should be plotted. Normally, this histogram has two peaks. The peak with a small degree of anisotropy represents the matrix, and the one with higher values corresponds to the fibres [33]. However, in our case only one peak was seen, which is related to the fibres. This means that there was no full-matrix voxel in our model. This could be due to the high fibre content (50 wt%) in the composite. As a result, no anisotropy filter is needed to be applied in the fibre orientation calculation. The principles of structure tensor analysis, which was implemented in VoxTex, for calculating the orientation distribution function and fibre orientation tensor, are explained in detail in Section 2.3 of [33].

**Figure 8:**
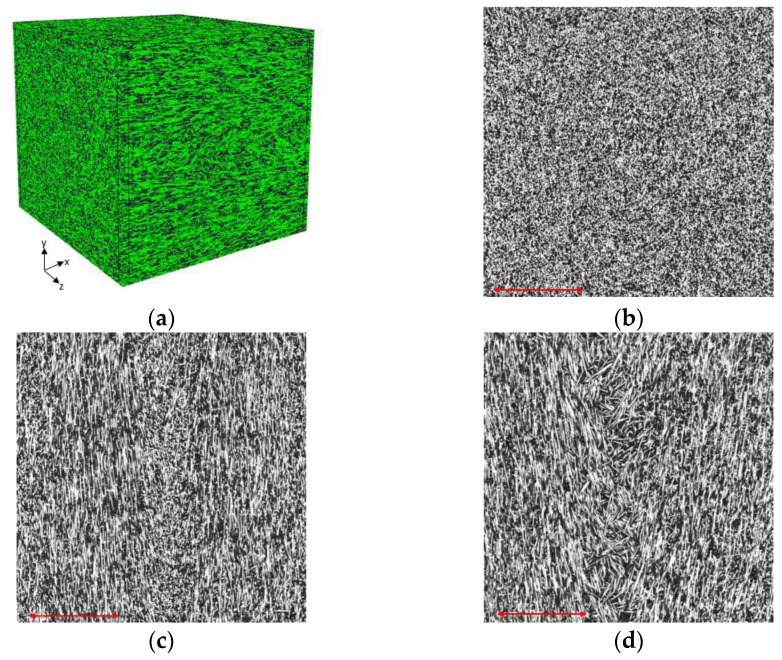
Miro-CT scans of the injection-moulded part: (**a**) a 3D view, (**b**) in y–z planes, (**c**) in x–z planes and (**d**) in x–y planes. (The scale bar in each figure shows a distance of 1 mm).

Figure 8 shows examples of micro-CT scans in different planes, which were taken of the centre of the ROI, and the reconstructed 3D image. It is evident that the majority of fibres were oriented along the flow direction (x-axis), as predicted by the Moldflow. For quantitative analysis, after generating the voxel model in VoxTex, the diagonal components of the FOT calculated by the VoxTex were compared to those obtained from the Moldflow simulation in the same ROI, and are represented in Table 2. Overall, the Moldflow FOT predictions were found to be highly consistent with the experimentally measured FOTs. These findings, which are in accordance with the previous studies [25,26], demonstrate that the Moldflow fibre orientation predictions can be trusted for finding the homogenised ETC.

#### 3.4.2. Homogenisation of Thermal Conductivity

Regarding the use of Giordano´s model to predict the ETC, a MATLAB script was written to select elements that comply with the transverse isotropy conditions. It is seen that not all elements associated with each zone will satisfy these conditions. The percentage of a number of elements satisfying the transverse isotropy conditions in each region can be used as a criterion for the validity of using Giordano´s model for predicting the ETC in that region of the part. This is indicated by %Ele. in Table 3. After calculating the parameter S for each valid element, the longitudinal and transversal ETC are computed for each element using Equations (1) and (2), and then can be volume averaged for the region of interest. In the two-step homogenisation approach, firstly $k_{x}=0.498$ and $k_{z}=0.386\;{\mathrm{Wm}}^{-1}K^{-1}$ were used in Equation (23) based on the Lielens prediction for unidirectional fibre alignment, according to Table 1. Afterwards, knowing the FOT for each element from the Moldflow analysis and using Equation (23), the orientation averaged ETC was computed along each direction for every element and then volume-averaged for the region. These data are reported in Table 3 and compared to the measured ETC obtained from experiments.

First of all, Giordano’s predictions can be implicitly trusted when the majority of elements associated with a region of interest satisfy the transverse isotropy conditions. This can be seen only in region B, since about two-third of elements comply with those conditions. Giordano’s predictions in this region agree well with that of the two-step homogenisation approach and the experimental measurements. On the contrary, in region A, no element satisfies the conditions. Hence, Giordano’s model failed to predict the ETC in this zone. Taking the values of diagonal components of the FOT in those regions into account, it can be deduced that Giordano’s model can be used when at least two diagonal components of the FOT are identical; that is, the fibre orientation distribution is symmetric around one axis, which is normally the flow direction in injection moulding. This could occur in situations where the orientation of fibres slightly differs from that of the perfectly aligned fibres, as this is the case for region B. As a result, it can be concluded that Giordano’s approach can be used in the ETC prediction of injection-moulded SFRPs when we are dealing with slightly-perturbed aligned orientations or a weakly expressed orientational preference [12]. These terms are used to describe orientation distributions that are close to perfectly aligned or completely uniform fibre distributions, respectively.

Overall, the predicted and measured ETC along each direction is proportional to the average values of the component of the FOT related to that direction. For instance, the average $a_{xx}$ is the highest in region B, followed by A and C. The same trend was also seen for modelled and measured $\langle k_{xx}\rangle$. Moreover, although there is generally good agreement between the predicted values of ETC along each direction and that of experimental measurements, it seems that the following trends existed for three zones:

$$\langle k_{xx}\rangle {}_{Experiment}<\langle k_{xx}\rangle {}_{modelling}\;\langle k_{yy}\rangle {}_{Experiment}>\langle k_{yy}\rangle {}_{modelling}\;\langle k_{zz}\rangle {}_{Experiment}>\langle k_{zz}\rangle {}_{modelling}\;$$

These trends could be explained by the fact that the MTPS method used for experimental measurement is not a purely one-dimensional thermal conductivity characterisation technique, even though it is an appropriate transient method for thermal conductivity measurement of an anisotropic material. In other words, when the ETC is measured along a particular axis normal to the plane which is in contact with the MTPS sensor, other in-plane components may play a role as well. For example, when measuring along the x-axis (the flow direction), along which the ETC is maximum in all three regions, interfering with the other two lower components of the ETC may decrease the measured $\langle k_{xx}\rangle$ compared to actual values, so $\langle k_{xx}\rangle {}_{Experiment}<\langle k_{xx}\rangle {}_{modelling}$. Conversely, measured $\langle k_{yy}\rangle$ and $\langle k_{zz}\rangle$ could be overestimated because of the influence of $\langle k_{xx}\rangle$ in measurements along y- and z-axes.

## 4. Conclusions

Two different homogenisation approaches based on the dielectric theory for pseudo-oriented inclusions (Giordano’s model) and the mean-field homogenisation scheme (the two-step homogenisation method) were used to predict the ETC of an injection-moulded SFRP. When unidirectional fibre alignment or uniformly distributed fibres are assumed, both models predict nearly identical ETCs which are compatible with FEM homogenisation results. For predicting the ETCs in the case of fibres oriented according to a specific FOT, three regions with different fibre orientations were considered in an injection-moulded tensile bar. The injection-packing process was firstly simulated via Autodesk Moldflow^®^ in order to obtain the FOT of elements associated with each region. By taking micro-CT images and processing the scans in the VoxTex software, it was discovered that the Moldflow FOT predictions and the experimentally obtained FOTs are very compatible.

A MATLAB script was written to find elements whose FOTs satisfy the transverse isotropy criteria, as required for Giordano’s model. Regarding the two-step homogenisation, an interpolative model based on Mori–Tanaka was used in the first step to predicting the ETC of unidirectional fibre alignment, followed by the orientation averaging in the second step. It was found that Giordano’s model can be used only in the gauge length of the sample. In this region, the fibre orientation distribution differs slightly from the unidirectional alignment. However, in other regions where the fibre orientation distribution noticeably deviates from either unidirectional or random orientations, the transverse isotropy conditions cannot be satisfied. Consequently, only the two-step homogenisation predictions are valid in these regions. Overall, experimental measurements were found to be in good agreement with the predicted values. However, while the $\langle k_{xx}\rangle$ measurements were marginally underestimated, the measured $\langle k_{yy}\rangle$ and $\langle k_{zz}\rangle$ seemed to be slightly higher than those of predictions. This observation can be explained by the fact that thermal conductivity measurements via the MTPS sensor are not exclusively unidirectional.

## Figures and Tables

**Table 1:** Comparison between longitudinal and transversal thermal conductivities obtained from Giordano, Mori–Tanaka and Lielens interpolation models to those of FEM homogenisation for unidirectional fibre alignment.

Model	Case Study 1: Random Orientation of the Fibres		Case Study 2: An Injection-Moulded SFRP	
	$k_{x}{\;}^{1}$	$k_{z}$	$k_{x}$	$k_{z}$
**Giordano**	67.96	66.67	0.498	0.384
**Mori–Tanaka**	67.96	66.70	0.498	0.378
**Lielens**	67.96	66.67	0.498	0.386
**FEM**	67.69	66.52	0.505	0.390

^1^ ETCs are reported in ${\mathrm{Wm}}^{-1}K^{-1}$.

**Table 2:** Comparison between the simulated and experimentally measured FOT in the gauge length of the injection-moulded part.

FOT	Moldflow Simulation	Micro-CT Scans Processed in VoxTex
$\mathrm{Average}\;a_{xx}$	0.85	0.87
$\mathrm{Average}\;a_{yy}$	0.09	0.09
$\mathrm{Average}\;a_{zz}$	0.06	0.04

**Table 3:** Comparison between predicted and measured ETCs along different directions for the injection-moulded SFRP.

Region	Avg. $a_{xx}$	Avg. $a_{yy}$	Avg. $a_{zz}$	Two-Step Hom.			Giordano			Experiments		
				$\langle k_{xx}\rangle$	$\langle k_{yy}\rangle$	$\langle k_{zz}\rangle$	% Ele.	$\langle k_{xx}\rangle$	$\langle k_{zz}\rangle$	$\langle k_{xx}\rangle$	$\langle k_{yy}\rangle$	$\langle k_{zz}\rangle$
**A**	0.63	0.35	0.02	0.457	0.425	0.388	0	-	-	0.452 ± 0.008	0.446 ± 0.007	0.409 ± 0.002
**B**	0.84	0.09	0.07	0.480	0.396	0.394	67%	0.487	0.394	0.472 ± 0.004	0.419 ± 0.005	0.418 ± 0.001
**C**	0.49	0.31	0.20	0.441	0.421	0.408	10%	0.463	0.409	0.441 ± 0.009	0.431 ± 0.004	0.424 ± 0.002

## Data Availability

Not applicable.

## Supplementary Materials

The following supporting information can be downloaded at: https://www.mdpi.com/article/10.3390/polym14163360/s1. Figure S1: The geometry of the injection-moulded part with the sprue and runners. Table S1: Injection moulding processing parameters.

## References

[1] Amjadi M., Fatemi A. (2021). A Fatigue Damage Model for Life Prediction of Injection-Molded Short Glass Fiber-Reinforced Thermoplastic Composites. Polymers.

[2] Huang C.-T., Chen X.-W., Fu W.-W. (2019). Investigation on the Fiber Orientation Distributions and Their Influence on the Mechanical Property of the Co-Injection Molding Products. Polymers.

[3] Naili C., Doghri I., Kanit T., Sukiman M.S., Aissa-Berraies A., Imad A. (2020). Short Fiber Reinforced Composites: Unbiased Full-Field Evaluation of Various Homogenization Methods in Elasticity. Compos. Sci. Technol..

[4] Wieme T., Duan L., Mys N., Cardon L., D’hooge D.R. (2019). Effect of Matrix and Graphite Filler on Thermal Conductivity of Industrially Feasible Injection Molded Thermoplastic Composites. Polymers.

[5] Zhai S., Zhang P., Xian Y., Zeng J., Shi B. (2018). Effective Thermal Conductivity of Polymer Composites: Theoretical Models and Simulation Models. Int. J. Heat Mass Transf..

[6] Tian W., Qi L., Zhou J., Liang J., Ma Y. (2015). Representative Volume Element for Composites Reinforced by Spatially Randomly Distributed Discontinuous Fibers and Its Applications. Compos. Struct..

[7] Sliseris J., Yan L., Kasal B. (2016). Numerical Modelling of Flax Short Fibre Reinforced and Flax Fibre Fabric Reinforced Polymer Composites. Compos. Part B Eng..

[8] Zhang N., Gao S., Song M., Chen Y., Zhao X., Liang J., Feng J. (2022). A Multiscale Study of CFRP Based on Asymptotic Homogenization with Application to Mechanical Analysis of Composite Pressure Vessels. Polymers.

[9] Mirkhalaf S.M., Eggels E., Anantharanga A.T., Larsson F., Fagerström M. (2019). Short Fiber Composites: Computational Homogenization vs Orientation Averaging. ICCM22.

[10] Duan H.L., Karihaloo B.L. (2007). Effective Thermal Conductivities of Heterogeneous Media Containing Multiple Imperfectly Bonded Inclusions. Phys. Rev. B.

[11] Kushch V.I., Sevostianov I. (2016). Maxwell Homogenization Scheme as a Rigorous Method of Micromechanics: Application to Effective Conductivity of a Composite with Spheroidal Particles. Int. J. Eng. Sci..

[12] Duan H.L., Karihaloo B.L., Wang J., Yi X. (2006). Effective Conductivities of Heterogeneous Media Containing Multiple Inclusions with Various Spatial Distributions. Phys. Rev. B.

[13] Ordóñez-Miranda J., Alvarado-Gil J.J., Medina-Ezquivel R. (2010). Generalized Bruggeman Formula for the Effective Thermal Conductivity of Particulate Composites with an Interface Layer. Int. J. Thermophys..

[14] Giordano S. (2003). Effective Medium Theory for Dispersions of Dielectric Ellipsoids. J. Electrostat..

[15] Giordano S. (2005). Order and Disorder in Heterogeneous Material Microstructure: Electric and Elastic Characterisation of Dispersions of Pseudo-Oriented Spheroids. Int. J. Eng. Sci..

[16] Pierard O., Friebel C., Doghri I. (2004). Mean-Field Homogenization of Multi-Phase Thermo-Elastic Composites: A General Framework and Its Validation. Compos. Sci. Technol..

[17] Tian W., Qi L., Su C., Zhou J., Jing Z. (2016). Numerical Simulation on Elastic Properties of Short-Fiber-Reinforced Metal Matrix Composites: Effect of Fiber Orientation. Compos. Struct..

[18] Tian W., Fu M.W., Qi L., Ruan H. (2020). Micro-Mechanical Model for the Effective Thermal Conductivity of the Multi-Oriented Inclusions Reinforced Composites with Imperfect Interfaces. Int. J. Heat Mass Transf..

[19] Camacho C.W., Tucker C.L., Yalvaç S., McGee R.L. (1990). Stiffness and Thermal Expansion Predictions for Hybrid Short Fiber Composites. Polym. Compos..

[20] Breuer K., Stommel M., Korte W. (2019). Analysis and Evaluation of Fiber Orientation Reconstruction Methods. J. Compos. Sci..

[21] Tian W., Qi L., Su C., Liang J., Zhou J. (2016). Numerical Evaluation on Mechanical Properties of Short-Fiber-Reinforced Metal Matrix Composites: Two-Step Mean-Field Homogenization Procedure. Compos. Struct..

[22] Modniks J., Andersons J. (2010). Modeling Elastic Properties of Short Flax Fiber-Reinforced Composites by Orientation Averaging. Comput. Mater. Sci..

[23] Mirkhalaf S.M., Eggels E.H., van Beurden T.J.H., Larsson F., Fagerström M. (2020). A Finite Element Based Orientation Averaging Method for Predicting Elastic Properties of Short Fiber Reinforced Composites. Compos. Part B Eng..

[24] Advani S.G., Tucker C.L. (1987). The Use of Tensors to Describe and Predict Fiber Orientation in Short Fiber Composites. J. Rheol..

[25] Caton-Rose P., Hine P., Bernasconi A., Conrado E. Experimental and Numerical Analysis of Fibre Orientation in Injection Moulded Short Glass Fibre Reinforced Polyamide 6 Notched Specimens. Proceedings of the 16th European Conference on Composite Materials.

[26] Foss P.H., Tseng H., Snawerdt J., Chang Y., Yang W., Hsu C. (2014). Prediction of Fiber Orientation Distribution in Injection Molded Parts Using Moldex3D Simulation. Polym. Compos..

[27] Tucker C.L., Liang E. (1999). Stiffness Predictions for Unidirectional Short-Fiber Composites: Review and Evaluation. Compos. Sci. Technol..

[28] Hiroshi H., Minoru T. (1986). Equivalent Inclusion Method for Steady State Heat Conduction in Composites. Int. J. Eng. Sci..

[29] Lielens G., Pirotte P., Couniot A., Dupret F., Keunings R. (1998). Prediction of Thermo-Mechanical Properties for Compression Moulded Composites. Compos. Part A Appl. Sci. Manuf..

[30] Whiteside B.R., Coates P.D., Hine P.J., Duckett R.A. (2000). Glass Fibre Orientation within Injection Moulded Automotive Pedal: Simulation and Experimental Studies. Plast. Rubber Compos..

[31] Fu S., Mai Y. (2003). Thermal Conductivity of Misaligned Short-fiber-reinforced Polymer Composites. J. Appl. Polym. Sci..

[32] Sinchuk Y., Finazzi D., Sevenois R., Van Paepegem W. (2022). Automatic instance segmentation of individual fibres in a short fibre glass/PA-6 composite. In *UGent Internal Report for FWO-SBO Project “Moccha-CT”*.

[33] Karamov R., Martulli L.M., Kerschbaum M., Sergeichev I., Swolfs Y., Lomov S.V. (2020). Micro-CT Based Structure Tensor Analysis of Fibre Orientation in Random Fibre Composites versus High-Fidelity Fibre Identification Methods. Compos. Struct..

[34] Straumit I., Lomov S.V., Wevers M. (2015). Quantification of the Internal Structure and Automatic Generation of Voxel Models of Textile Composites from X-ray Computed Tomography Data. Compos. Part A Appl. Sci. Manuf..

[35] Stratton J.A. (2007). Electromagnetic Theory.

