# Supplementary material for: Homogenisation of the Local Thermal Conductivity in Injection-Moulded Short Fibre Reinforced Composites
Source: Polymers (Basel). 2022 Aug 17;14(16):3360. doi: 10.3390/polym14163360 (PMC9415741; doi:10.3390/polym14163360)
Supplement: Supplementary file 1 [file polymers-14-03360-s001.zip › polymers-1797982-supplementary.pdf]

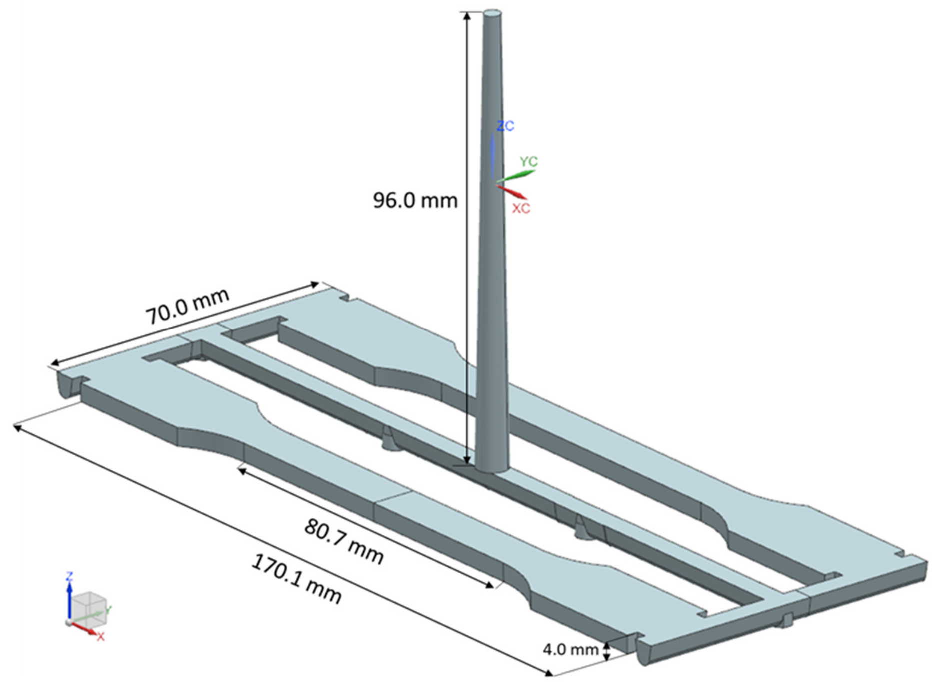

Figure S1. The geometry of the injection moulded part with the sprue and runners.

Table S1. Injection moulding processing parameters.

**Process Parameters**	**Values**
Melt Temperature	270 °C
Packing pressure	35 MPa
Packing time	10 sec
Injection speed	20 cm^3/sec
Cooling time	30 sec
Mould temperature	80 °C
